# Intense genomic reorganization in the genus *Oecomys* (Rodentia, Sigmodontinae): comparison between DNA barcoding and mapping of repetitive elements in three species of the Brazilian Amazon

**DOI:** 10.3897/CompCytogen.v10i3.8306

**Published:** 2016-09-08

**Authors:** Renan Gabriel Gomes Júnior, Carlos Henrique Schneider, Thatianna de Lira, Natália Dayane Moura Carvalho, Eliana Feldberg, Maria Nazareth Ferreira da Silva, Maria Claudia Gross

**Affiliations:** 1 Universidade Federal do Amazonas, Instituto de Ciências Biológicas, Departamento de Genética, Laboratório de Citogenômica Animal, Av. General Rodrigo Otávio, 3000, Japiim, Zip code 69077-000 Manaus, AM, Brazil; 2 Instituto Nacional de Pesquisas da Amazônia, Av. André Araújo, 2936 Zip Code 69077-000, Manaus, AM, Brazil; 3 Universidade Federal da Integração Latino Americana, Laboratório de Genética, Av. Tarquínio Joslin dos Santos, 1000, Jardim Universitário, Zip code 85857-190, Foz do Iguaçu, PR, Brazil

**Keywords:** Oryzomyini, FISH, telomere, rDNA, heterochromatin, COI

## Abstract

*Oecomys* Thomas, 1906 is one of the most diverse and widely distributed genera within the tribe Oryzomyini. At least sixteen species in this genus have been described to date, but it is believed this genus contains undescribed species. Morphological, molecular and cytogenetic study has revealed an uncertain taxonomic status for several *Oecomys* species, suggesting the presence of a complex of species. The present work had the goal of contributing to the genetic characterization of the genus *Oecomys* in the Brazilian Amazon. Thirty specimens were collected from four locations in the Brazilian Amazon and three nominal species recognized: *Oecomys auyantepui* (Tate, 1939), *Oecomys bicolor* (Tomes, 1860) and *Oecomys rutilus* (Anthony, 1921). COI sequence analysis grouped *Oecomys auyantepui*, *Oecomys bicolor* and *Oecomys rutilus* specimens into one, three and two clades, respectively, which is consistent with their geographic distribution. Cytogenetic data for *Oecomys auyantepui* revealed the sympatric occurrence of two different diploid numbers, 2n=64/NFa=110 and 2n=66/NFa=114, suggesting polymorphism while *Oecomys bicolor* exhibited 2n=80/NFa=142 and *Oecomys rutilus* 2n=54/NFa=90. The distribution of constitutive heterochromatin followed a species-specific pattern. Interspecific variation was evident in the chromosomal location and number of 18S rDNA loci. However, not all loci showed signs of activity. All three species displayed a similar pattern for 5S rDNA, with only one pair carrying this locus. Interstitial telomeric sites were found only in *Oecomys auyantepui*. The data presented in this work reinforce intra- and interspecific variations observed in the diploid number of *Oecomys* species and indicate that chromosomal rearrangements have led to the appearance of different diploid numbers and karyotypic formulas.

## Introduction

The order Rodentia is divided into nine taxonomic families in Brazil. The family Cricetidae contains the most members, among which the subfamily Sigmodontinae includes 86 genera and 395 species (*sensu* Reig 1980) according to Prado and Percequillo (2013). Oryzomyini is the most diverse tribe of the Sigmodontinae, and the genus *Oecomys* Thomas, 1906 is one of the most diverse of the tribe Oryzomyini (Prado and Percequillo 2013). However, its morphological and karyological distinction and generic status were only recognized relatively recently (Andrades-Miranda et al. 2001, Carleton and Musser 1984, Gardner and Patton 1976, Reig 1984, 1986 as cited in Musser and Carleton 2005). Similarity among species and the limited understanding of morphological variations in *Oecomys* (including interspecific, intraspecific, geographic, and specimen age-inherent variations) have rendered species identification difficult.

Currently, 16 species are recognized within this genus (Musser and Carleton 2005, Carleton et al. 2009), but only nine species have been studied for karyotypes, showing 11 different diploid numbers, varying between 54 and 86 chromosomes (Table 1). In Brazil 12 species were registered and 9 of which can be found in Amazon biome; *Oecomys auyantepui* Tate, 1939, *Oecomys bicolor* (Tomes, 1860), *Oecomys concolor* (Wagner, 1845), *Oecomys paricola* (Thomas, 1904), *Oecomys rex* Thomas, 1910, *Oecomys roberti* (Thomas, 1904), *Oecomys rutilus* Anthony, 1921, *Oecomys superans* Thomas, 1911 and *Oecomys trinitatis* (J. A. Allen & Chapman, 1893) (Bonvicino et al. 2008; Flores 2010). Variations in fundamental number have also been reported in species with the same diploid number, which is an indicator of chromosomal rearrangements within the group (Rosa et al. 2012). However, morphological and morphometric analysis in conjunction with molecular and cytogenetic approaches revealed uncertainty in the delimitation and distribution of *Oecomys* species, suggesting the presence of a complex of species (Patton and Sherwood 1983, Emmons and Feer 1997, Patton et al. 2000, Musser and Carleton 2005, Carleton et al. 2009, Flores 2010, Rosa et al. 2012).

**Table 1.** Karyotypes recorded for species of the genus *Oecomys*. Diploid Number (2n), fundamental number (FN) and location are listed.

Species	Location	2n	FN	Reference
*Oecomys auyantepui*	Jari river – PA	72	80	Lira (2012)
*Oecomys auyantepui*	Jatapu river – AM	64 66	110 114	Present paper Present paper
Oecomys bahienses**	São Lourenço da Mata – PE	60	62	Langguth et al. (2005)
*Oecomys bicolor*	Jari river – PA	54	82	Lira (2012)
*Oecomys bicolor*	SUR	80	–	Baker et al. (1983)
*Oecomys bicolor*	RR Ipameri and Serra da mesa– GO	80	124	Andrades-Miranda et al. (2000) Andrades-Miranda et al. (2001)
*Oecomys bicolor*	Curanja river – PER	80	134	Gardner and Patton (1976)
*Oecomys bicolor*	Curanja river – PER	80	136	Gardner and Patton (1976)
*Oecomys bicolor*	Juruá river – AM	80	140	Patton et al. (2000)
*Oecomys bicolor*	Purus and Jatapu river – AM	80	142	Present paper
*Oecomys bicolor*	? Hydropower plant UEH Samuel – GO	82	110	Andrades-Miranda et al. (2000) Andrades-Miranda et al. (2001)
*Oecomys bicolor*	Jari river – PA	82	116	Lira (2012)
*Oecomys bicolor*	Jurua river – AM	86	98	Patton et al. (2000)
*Oecomys catherinae*	GO, São Lourenço da Mata – PE Ubatuba – SP, Cruz do Espírito Santo – PB, Igarassú, Jaqueira and Paudalho – PE RJ	60	62	Andrades-Miranda et al. (2001) Andrade and Bonvicino (2003) Langguth et al. (2005) Pinheiro and Geise (2008) Asfora et al. (2011)
*Oecomys catherinae*	Ubatuba – SP RJ	60	64	Pinheiro e Geise (2008) Asfora et al. (2011)
*Oecomys catherinae*	RJ, SP	86	98	Patton et al. (2000)
*Oecomys concolor*	PAN	58	–	Baker et al. (1983)
*Oecomys concolor*	SUR	60	–	Baker et al. (1983)
*Oecomys concolor*	Villavicencio – COL	60	62	Gardner and Patton (1976)
*Oecomys concolor*	MEX	60	–	Andrade and Bonvicino (2003)
*Oecomys concolor*	MEX	61	–	Andrade and Bonvicino (2003)
*Oecomys concolor*	Curanja River – PER	80	112	Gardner and Patton (1976)
*Oecomys concolor*	DF, RJ, GO, SP, RO	60	62	Gardner and Patton (1976) Svartman (1989) Andrades-Miranda et al. (2000) Andrades-Miranda et al. (2001) Andrade and Bonvicino (2003)
*Oecomys paricola*	Environment Park – PA	68	72	Rosa et al. (2012)
*Oecomys paricola*	Marajó island – PA	70	72	Rosa et al. (2012)
*Oecomys paricola*	Environment Park – PA	70	76	Rosa et al. (2012)
*Oecomys rex*	Jari river – PA	62	80	Lira (2012)
*Oecomys roberti*	AM	80	114	Patton et al. (2000)
*Oecomys roberti*	Juruá river – AM Jamari river – RO	82	106	Langguth et al. (2005)
*Oecomys rutilus*	Negro river – AM	54	90	Present paper
*Oecomys superans*	PER Jurua river – AM	80	108	Gardner and Patton (1976) Andrade and Bonvicino (2003) Patton et al. (2000)
*Oecomys trinitatis*	Jurua river – AM	58	96	Patton et al. (2000)
*Oecomys* sp.	Cuieiras river – AM	54	84	Lira (2012)
*Oecomys* sp.	Jatapu – AM	54	86	Lira (2012)
*Oecomys* sp.	MS	72	90	Andrade and Bonvicino (2003)

*The location indicates the sampled countries or Brazilian states. AM = Amazonas , GO = Goiás , MS = Mato Grosso do Sul , PA = Pará , PB = Paraíba , PE = Pernambuco , RJ = Rio de Janeiro , RO = Rondônia , RR = Roraima , SP = São Paulo , COL = Colombia , MEX = Mexico , PAN = Panama , PER = Peru , SUR = Suriname . **Synonym of *Oecomys catherinae*.

Hence, in the present study, we used classic and molecular cytogenetics approaches in order to enable the genetic characterization of three species of the genus *Oecomys* from the Brazilian Amazon. Further, we used DNA barcoding to evaluate the intra- and interspecific distances, and infer the utility in species identification by combining this dataset with sequences deposited in GenBank.

## Materials and methods

### Samples

**Figure 1.**
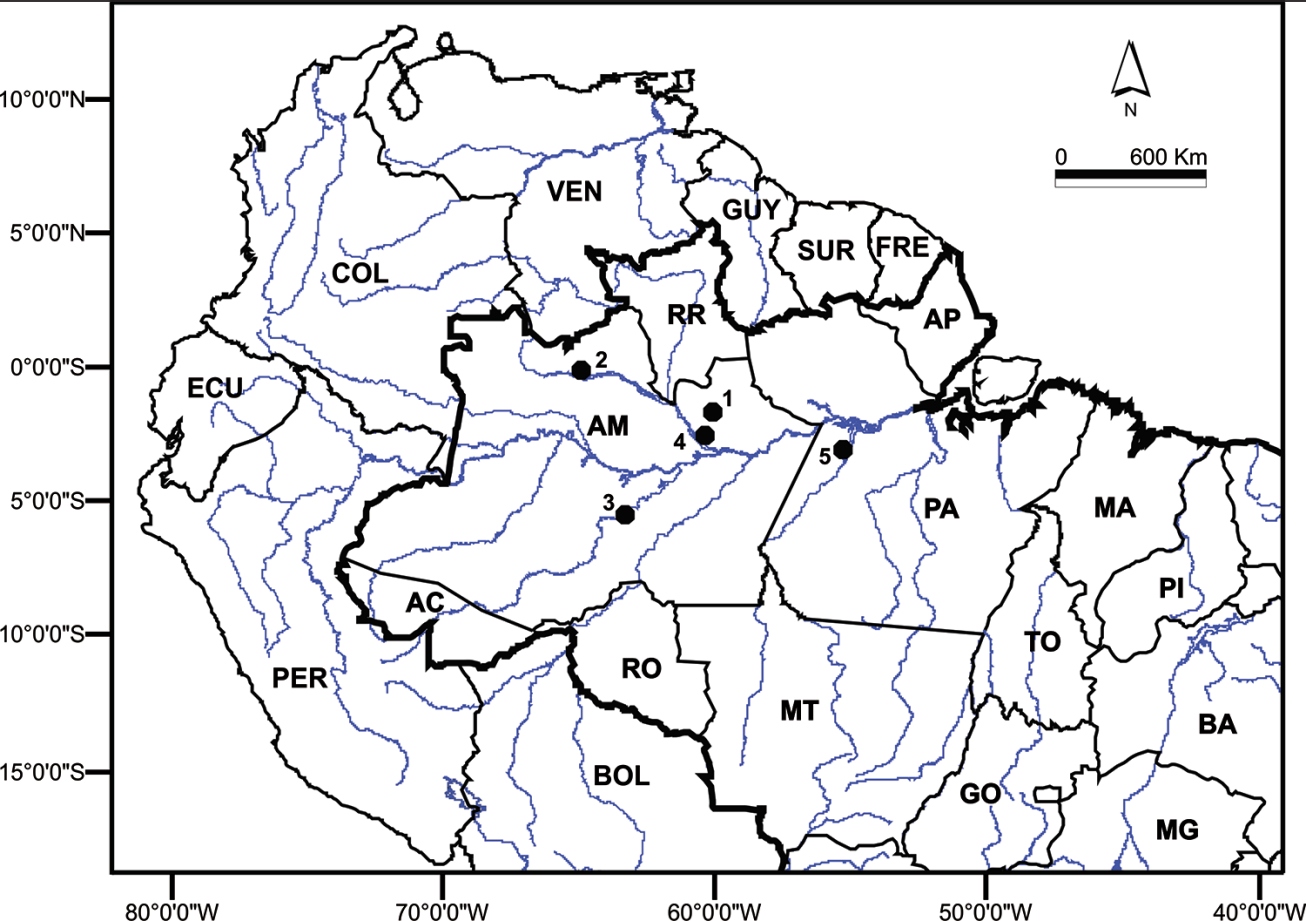
Map of the Brazilian Amazon, indicating the collection sites. The left and right banks of the following Amazonas state rivers were sampled: **1** Jatapú (near the city of São Sebastião do Uatumã - 0°50’ to 1°55'S; 58°50’ to 60°10'W) **2** Negro (near the city of Santa Isabel do Rio Negro - 0°24.4'N; 65°1.017'W) **3** Purus (near the city of Tapauá - 05°42.183'S, 63°13.967'W) **4** Cuieiras (02°47'S, 60°27'W) **5** Tapajós (03°21.283'S, 55°11.733'W). BOL = Bolivia, PER = Peru, ECU = Ecuador, COL = Colombia, VEN = Venezuela, GUY = Guyana, SUR = Suriname, FRE = French Guyana, RR = Roraima, AP = Amapa, AM = Amazonas, PA = Para, RO = Rondonia, AC = Acre, MA = Maranhão, PI = Piaui, TO = Tocantins, BA = Bahia, MT = Mato Grosso, GO = Goias, MG = Minas Gerais.

Thirty specimens were collected from five locations in the Brazilian Amazon (Fig. 1, Table 2) and euthanized according to the recommendations of Resolution CFBIO N. 301 from December 8^th^, 2012. Voucher specimens were prepared or fixed, and stored in 70% ethanol; the specimens are currently stored in the mammal collection of the National Institute of Amazonian Research [Instituto Nacional de Pesquisas da Amazônia – INPA] (Table 2). The methods for the collection, maintenance and processing of the material complied with the guidelines of the Brazilian College of Animal Experimentation [Colégio Brasileiro de Experimentação Animal – COBEA] and were approved by the Ethics Committee On Animal Use of the Federal University of Amazonas [Comissão de Ética no Uso de Animais da Universidade Federal do Amazonas] (043/2013-CEUA/UFAM). Individuals were collected with the permission of the Chico Mendes Institute for Biodiversity Conservation [(Instituto Chico Mendes de Conservação da Biodiversidade – ICMBIO), License No. 10832-1 /35513-1). It must be noted that the collections took place outside of conservation units and that these species are not threatened with extinction. Samples were collected from the hematopoietic organ of each individual following euthanasia to obtain chromosome preparations and muscle tissue for DNA extraction.

**Table 2.** Species of *Oecomys* collected in present work: The voucher, collection sites, sex, diploid number (2n), fundamental number (FN), karyotype formula, Nucleolus organizer region (NOR), rDNA 18S (18S), rDNA 5S (5s) are listed; M = male; F = female; m = metacentric; sm = submetacentric; st = subtelocentric; a = acrocentric; X = Sexual chromosome X; Y = Sexual chromosome Y. Bold voucher were karyotyped in the present work.

Species	Voucher	Sex	Collection sites	2n	FN	Karyotype formula	NOR	18S	5S
*Oecomys auyantepui*	**INPA 6754**	M	Brazil, AM – Jatapú River	64	110	12m+10sm+26st +16a+XY	10p and 14p	10p and 14p	5p
	**INPA 6751**	M		66	112	16m+6sm+26st+ 14a+XY			
	INPA 6753	M		–	–	–	–	–	–
	INPA 6747	M		–	–	–	–	–	–
*Oecomys bicolor*	**INPA 6772**	M	Brazil, AM – Purus River	80	142	18m+10sm+36st+ 14a+XY	15p, 18p, 21p, 22p and 26p	2p, 3p, 13p, 15p, 16p, 18p, 19p, 21p, 22p, 25p 26p and 30p	7p
	**INPA 6749**	M	Brazil, AM – Jatapú River						
	**INPA 6756**	M							
	**INPA 6758**	M							
	**INPA 6757**	F							
	INPA 6752	M	Brazil, AM – Jatapú River	–	–	–	–	–	–
	INPA 6773	F	Brazil, AM – Purus River	–	–	–	–	–	–
	INPA 6770	M	Brazil, AM – Negro River	–	–	–	–	–	–
	INPA 6775	M	Brazil, PA – Tapajós River	–	–	–	–	–	–
*Oecomys rutilus*	**INPA 6760**	F	Brazil, AM – Negro River	54	90	24m+6sm+8st+ 14a+XX	4p and 23p	4p and 23p	1p
	**INPA 6761**	F							
	**INPA 6762**	F							
	**INPA 6768**	M							
	INPA 6767	F		–	–	–	–	–	–
	INPA 6769	M		–	–	–	–	–	–
	INPA 6766	F		–	–	–	–	–	–
	INPA 6763	F		–	–	–	–	–	–
	INPA 6764	F		–	–	–	–	–	–
	INPA 6765	F		–	–	–	–	–	–
	INPA 6774	F		–	–	–	–	–	–
	INPA 6759	F		–	–	–	–	–	–
	INPA 6745	F	Brazil, AM – Cuieiras River	–	–	–	–	–	–
	INPA 6746	M		–	–	–	–	–	–
	INPA 6744	F		–	–	–	–	–	–
	INPA 6750	M	Brazil, AM – Jatapú River	–	–	–	–	–	–
	INPA 6755	M		–	–	–	–	–	–
	INPA 6748	F		–	–	–	–	–	–

### Chromosome analysis

Mitotic chromosomal preparations were obtained using the protocol described by Ford and Harmerton (1956), with some modifications. Nucleolus organizing regions (NORs), heterochromatin and G-banding were identified through silver nitrate staining (Howell and Black 1980), the C-banding technique (Sumner 1972) and trypsin solution (Seabright 1971), respectively. 5S and 18S rDNA probes were obtained after PCR amplification using the following primers: 5Sf (5’-CAG GGT CGG GCC TGG TTA GTA-3’) and 5Sr (5’-CTT CYG AGA TCA GAC GAG ATC-3’); 18Sf (5’-CCG CTT TGG TGA CTC TTG AT-3’) and 18Sr (5’-CCG AGG ACC TCA CTA AAC CA-3’) (Gross et al. 2010). For telomere sequences, DNA-free amplifications were performed using the primers (TTAGGG)_5_ and (CCCTAA)_5_ (Ijdo et al. 1991). Amplification reactions were conducted in a total volume of 25 µl (~100 ng of genomic DNA), containing 10× reaction buffer (final concentration: 10 mM Tris-HCl; 1.5 mM MgCl_2_; 50 mM KCl; pH 8.3), 0.3 units of Taq DNA polymerase, 0.2 mM each dNTP, 0.2 µl of each primer and Milli-Q water to the final volume; the annealing temperature was 56 ºC for 18S rDNA and 59 °C for 5S rDNA, and the final volume was 25 µl. The 5S gene PCR product was labeled with Biotin (Biotin Nick translations mix, Roch) and the 18S gene and telomere sequences with digoxigenin (Dig-Nick Translation mix, Roche), following the manufacturer’s instructions. Alexa Fluor 488-conjugated streptavidin (Life technologies) and anti-digoxigenin rhodamine (Roche) antibodies were used to detect the probe signal. Fluorescent *in situ* hybridization was carried out based on the protocols described by Pinkel et al. (1986).

Slides were screened for metaphases, at least 30 for each technique were analyzed and the best metaphases were photo-documented using an Olympus BX-51 epifluorescence microscope. Chromosomes were organized by decreasing size, and their morphology was determined based on the centromere position, being classified as metacentric (m), submetacentric (sm), subtelocentric (st) or acrocentric (a) (Levan et al. 1964).

### Mitochondrial DNA analysis

DNA was extracted according to the protocol described by Sambrook and Russel (2001). The cytochrome oxidase subunit I (COI) gene sequence was obtained through PageBreakPageBreak polymerase chain reaction (PCR) using the universal primers described by Ivanova et al. (2007). The PCR products were purified with the ExoSap® kit (GE Healthcare) and sequenced using the method described by Sanger et al. (1977) on an ABI 3130XL automatic sequencer. The resulting sequences were submitted to the NCBI database under the following accession numbers: KT258600–KT258632.

Sequences were manually aligned using BioEdit v7.2.2 software (Hall 2001) and compared with sequences deposited in GenBank using BLASTn (Basic Local Alignment Search Tool). A Bayesian phylogenetic analysis was conducted with MrBayes 3.2 (Ronquist and Huelsenbeck 2003). For this analysis, Markov Chain Monte-Carlo sampling was conducted every 20,000^th^ generation until the standard deviation of split frequencies was <0.01. A burn-in period equal to 25% of the total generations was required to summarize the parameter values and trees. Parameter values were assessed based on 95% credibility levels to ensure that the analysis had run for a sufficient number of generations. A genetic distance matrix was constructed using the MEGA 6 program (Tamura et al. 2013) and was obtained according to the Kimura 2 parameter (K2p) model. For Bayesian analysis, 53 *Oecomys* COI sequences available in GenBank were included (Appendix 1). One specimen of *Euryoryzomys macconnelli* was used as an outgroup.

## Results

### Chromosome analysis

#### *Oecomys auyantepui* – Jatapú River

**Figure 2.**
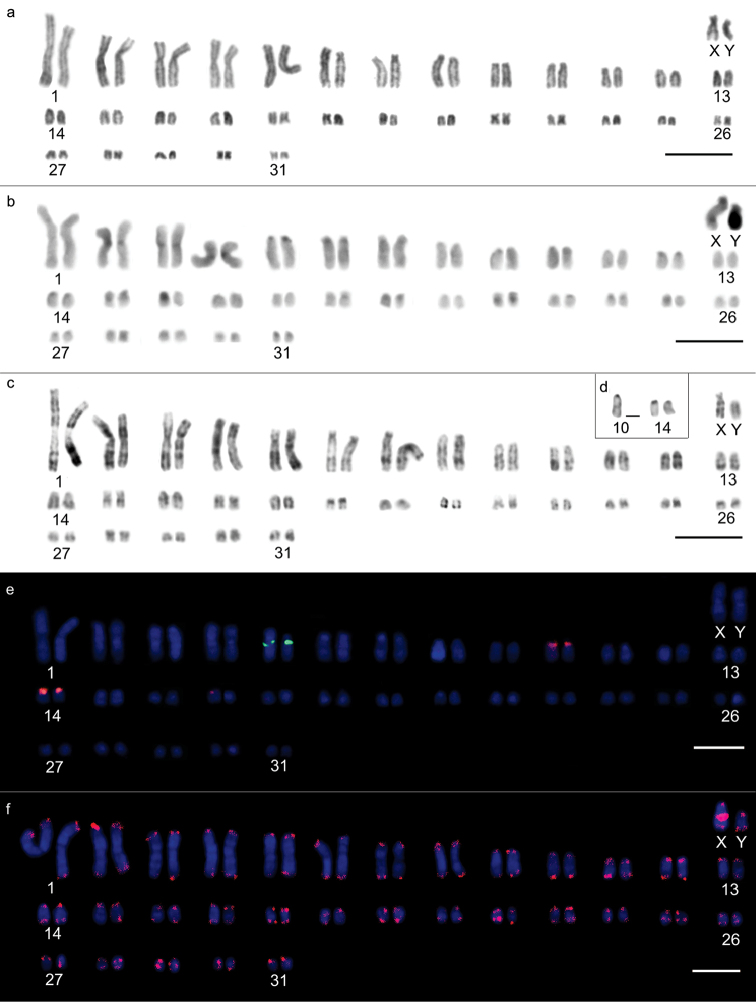
Karyotypic characteristics of male *Oecomys auyantepui*, karyomorph “a” (INPA 6754) with 2n=64: **a** conventional Giemsa staining **b** heterochromatic regions highlighted by C-banding **c** G-banding **d** nucleolus organizing region-carrying pairs evidenced by silver nitrate staining **e** fluorescent *in situ* hybridization of 5S rDNA (green) and 18S rDNA (red) probes **f** karyotype indicating the presence of telomeric sites as well as interstitial telomeric sequence in the sex X chromosome. Bars: 10 µm.

**Figure 3.**
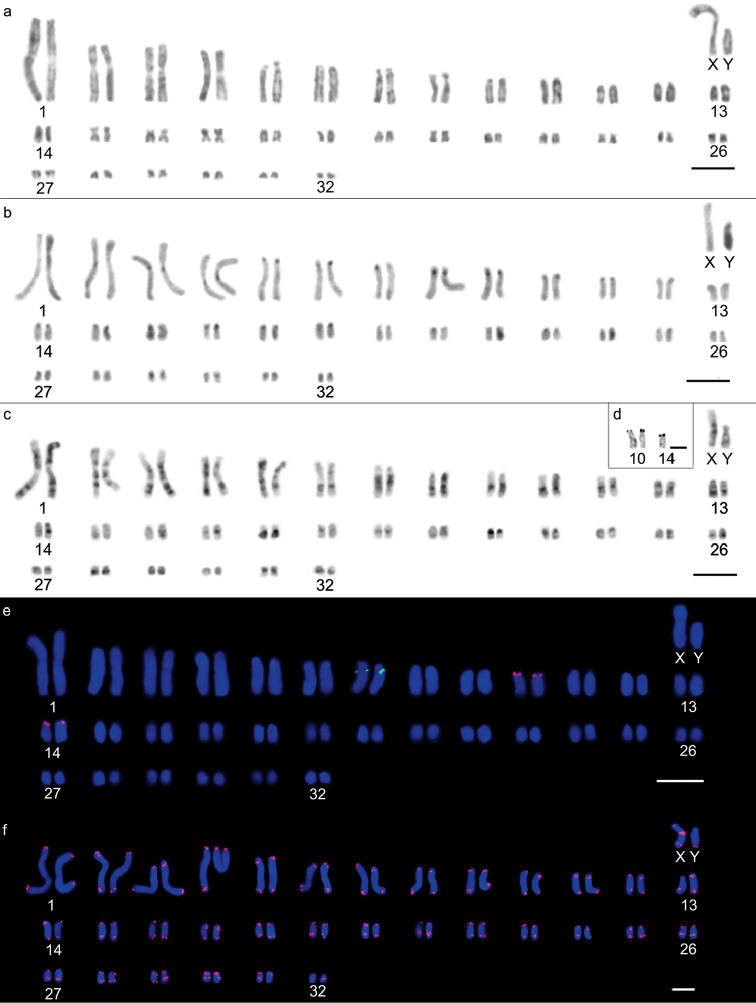
Karyotypic characteristics of male *Oecomys auyantepui* karyomorph “b”, with 2n=66: **a** conventional Giemsa staining (INPA 6751) **b** heterochromatic regions highlighted by C-banding (INPA 6751) **c** G-banding (INPA 6751) **d** nucleolus organizing region-carrying pairs revealed by silver nitrate staining (INPA 6751) **e** fluorescent *in situ* hybridization of 5S rDNA (green) and 18S rDNA (red) probes (INPA 6751) **f** karyotype indicating the presence of telomeric sites as well as an interstitial telomeric sequence on the X sex chromosome (INPA 6754). Bars: 10 µm.

Two different diploid numbers were observed along the same bank of the Jatapú River: Karyomorph “a” exhibited 2n=64 chromosomes, a fundamental number = 110, and a karyotypic formula of 16m+6sm+26st+14a+XY (Fig. 2), in which pairs 1, 4, 15, 22, 26, 28, 30 and 31 were metacentric; 2, 3 and 19 were submetacentric; 5-13, 23, 24, 25 and 27 were subtelocentric; and 14, 16, 17, 18, 20, 21 and 29 were acrocentric (Fig. 2a). Karyomorph “b” exhibited 2n=66 chromosomes, a fundamental number = 112 and a karyotypic formula of 12m+10sm+26st+16a+XY (Fig. 3), in which pairs 1, 4, 17, 21, 24 and 29 were metacentric; 2, 3, 15, 16 and 27 were submetacentric; 5, 6, 7, 8, 9, 12, 18, 19, 20, 23, 25, 26 and 28 were subtelocentric; and 10, 11, 13, 14, 22, 30, 31 and 32 were acrocentric (Fig. 3a). Chromosomes X and Y were submetacentric for 2n=64 (Fig. 2a), whereas for 2n=66, chromosome X was metacentric, and chromosome Y was submetacentric and half the size of chromosome X (Fig. 3a). The heterochromatin was predominantly centromeric for both 2n=64 and 2n=66 chromosomes, ranging between subtle and conspicuous (Figs 2b, 3b). The Y chromosome exhibited a heterochromatic long arm in both karyotypes, while the X chromosomes presented a centromeric block and bitelomeric labeling. G-banding patterns enabled the identification of homologous pairs for each karyomorph (Figures 2c, 3c) and homology detection among the largest pairs of the complement. Pairs 1, 2, 3, 4, 5, 6, 12 and 13 from the 2n=66 chromosome karyomorph were homologous to pairs 1, 3, 2, 4, 5, 6, 12 and 13 from the 2n=64 chromosome karyomorph, respectively. Silver nitrate staining of the 2n=64 karyomorph resulted in labeling of three terminal sites, on one of the chromosomes of pair 10 and on both of the pair 14 homologs (Fig. 2d). The 2n=66 karyomorph also exhibited labeling of three terminal sites, two on pair 10 and one on one of the pair 14 chromosomes (Fig. 3d).

18S rDNA loci were visualized on chromosome pairs 10 and 14 of both karyomorphs, while the single 5S rDNA loci was located on pair 5 of karyomorph “a” and pair 7 of karyomorph “b” (Figs 2e, 3e). Both karyomorphs presented interstitial telomeric sequences (ITSs) in the centromeric region of the X chromosome (Figs 2f and 3f).

#### *Oecomys bicolor* – Jatapú, Negro and Purus rivers

**Figure 4.**
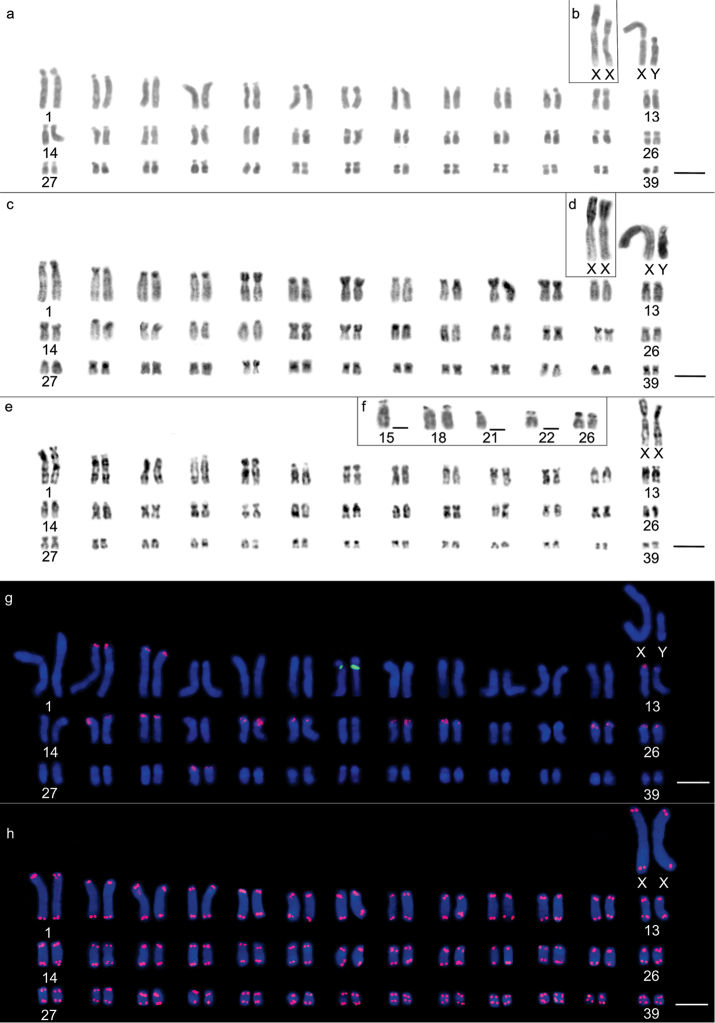
Karyotypic characteristics of *Oecomys bicolor*: **a** conventional Giemsa staining of a male (INPA 6749) **b** highlighted sex chromosomes of a female (INPA 6749) **c** heterochromatic regions revealed by C-banding in a female (INPA 6772) **d** highlighted C-banding on a male’s sex chromosomes (INPA 6772) **e** G-banding of a female (INPA 6772) **f** nucleolus organizing region-carrying pairs revealed by silver nitrate staining (INPA 6749) **g** fluorescent *in situ* hybridization of 5S rDNA (green) and 18S rDNA (red) probes (INPA 6758) **h** karyotype indicating the presence of telomeric sites (INPA 6772). Bars: 10 µm.

*Oecomys bicolor* was found to exhibit a diploid number 2n=80 chromosomes, a fundamental number = 142, and a karyotypic formula of 18m+10sm+36st+14a+XX or XY, wherein pairs 12, 32, 33, 34, 35, 36, 37, 38 and 39 were metacentric; pairs 7, 20, 25, 26 and 27 were submetacentric; 1, 2, 5, 6, 10, 11, 13, 14, 15, 16, 17, 19, 21, 22, 23, 24, 29 and 30 were subtelocentric; and 3, 4, 8, 9, 18, 28 and 31 were acrocentric (Figs 4a, 4b), with no differences being observed among individuals from the three collection sites. Sex chromosome X is the largest submetacentric chromosome of the complement, while sex chromosome Y is an average subtelocentric chromosome (Fig. 4b). Heterochromatin can be found in conspicuous blocks in the centromere region of all chromosomes, and in the case of the majority of metacentric, submetacentric (Fig. 4c), and the sex X chromosome, it also extends into the short arm (Fig. 4d). G-banding patterns enabled the correct identification of homologous pairs (Fig. 4e). Silver nitrate staining showed multiple terminal type-NORs on both homologous chromosomes of pairs 18 and 26 and on one of the homologous chromosome of pairs 15, 21 and 22 (Fig. 4f). 18S rDNA loci were identified on both homologous chromosomes of pairs 2, 3, 13, 15, 16, 18, 19, 21, 22, 25, 26 and 30, whereas 5S rDNA locus was located only on pair 7 (Fig. 4g). No ITSs could be observed (Fig. 4h).

#### *Oecomys rutilus* – Cuieiras, Jatapú and Negro rivers

**Figure 5.**
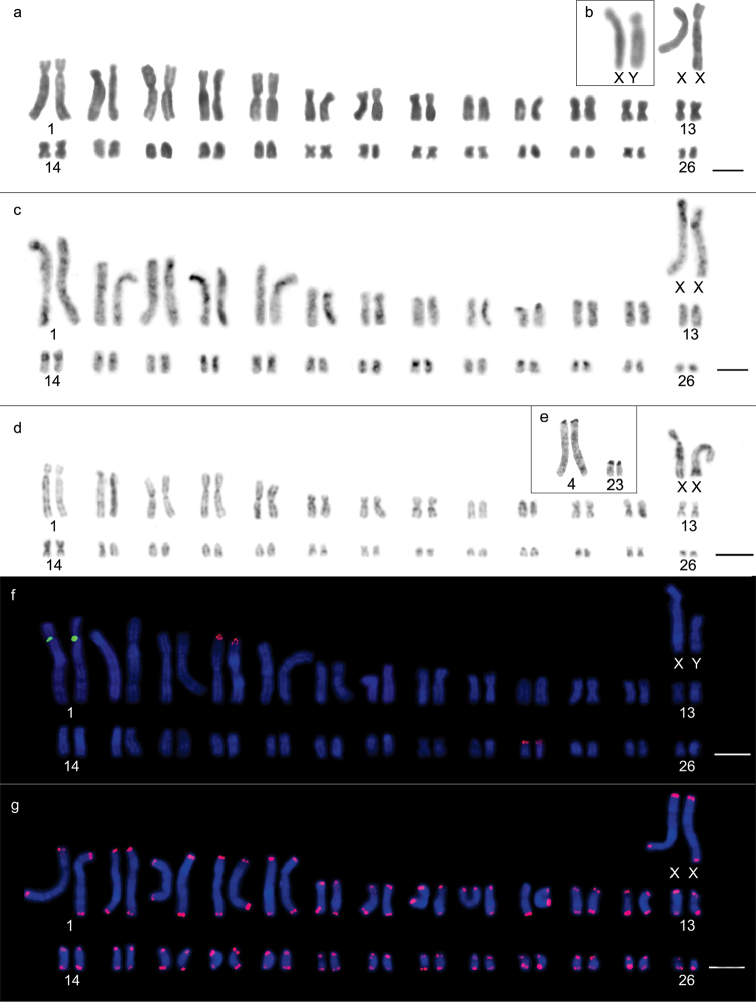
Karyotypic characteristics of *Oecomys bicolor*: **a** conventional Giemsa staining of a male (INPA 6761) **b** highlighted sex chromosomes of a male (INPA 6768) **c** heterochromatic regions revealed by C-banding of a male individual (INPA 6754) **d** G-banding of a female (INPA 6761) **e** nucleolus organizing region-carrying pairs revealed by silver nitrate staining (INPA 6762) **f** fluorescent *in situ* hybridization of 5S rDNA (green) and 18S rDNA (red) probes (INPA 6761) **g** karyotype indicating the presence of telomeric sites (INPA 6761). Bars: 10 µm.

*Oecomys rutilus* was characterized as showing a diploid number 2n=54 chromosomes and a fundamental number = 90, with a karyotypic formula 24m+6sm+8st+14a+XX or XY, in which pairs 3, 5, 11, 12, 13, 14, 15, 19, 21, 22, 25 and 26 were metacentric; 7, 8 and 20 were submetacentric; 1, 2, 4 and 6 were subtelocentric; and 9, 10, 16, 17, 18, 23 and 24 were acrocentric (Figs 5a, 5b), with no differences being detected between the specimens collected at three different sites. The X chromosome was large and submetacentric, while the Y chromosome was subtelocentric and approximately 3/4 of the size of chromosome X (Fig. 5b). Heterochromatic regions were characterized by subtle or conspicuous centromeric labeling on some chromosome pairs (Fig. 5c). G-banding patterns enabled correct homologous pairing (Fig. 5d). Multiple NORs were revealed by silver nitrate staining in the terminal regions of both homologous chromosomes of pairs 4 and 23 (Fig. 5e), coinciding with 18S rDNA loci (Fig. 5f), which were also observed on both homologous chromosomes of pair 1, in a proximal position on the long arms (Fig. 5f). No ITSs were observed (Fig. 5g).

### Mitochondrial DNA identification

**Figure 6.**
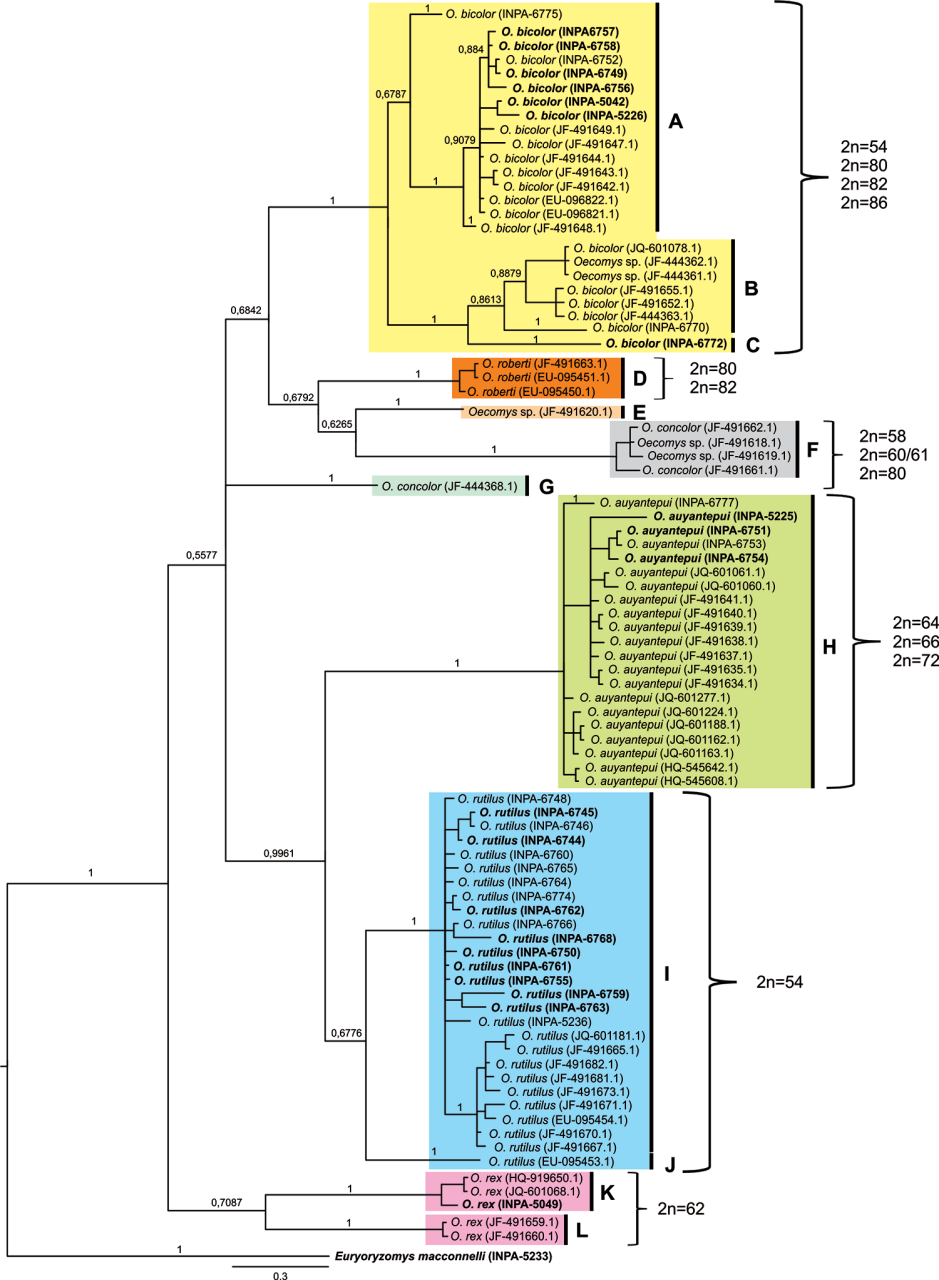
Bayesian tree of the cytochrome oxidase I gene. The probabilistic support is presented above the branches. Letters (**A–L**) represent the groups formed based on the analysis of the genetic distances between them. Sequences in bold were analyzed in the present work.

A total of 86 *Oecomys* mitochondrial COI gene sequences were compared: 33 originating from the present work and 53 deposited in GenBank (Appendix 1). NJ, Bayesian and ML tree retrieved the same topology and showed differences mainly in relation to branch support values. The similarity index was greater than 98%, which allowed molecular identification of the species. The phylogenetic trees (Figure 6) grouped *Oecomys rutilus* into two clades, one comprising individuals from Brazil, Suriname and Guyana (I), while the other consisted of one individual from Ecuador (J). The genetic distance between clades I and J was 7.33%, whereas the genetic distance within clade I was 1.62%. The individuals of *Oecomys auyantepui* were grouped into a single clade (H), comprising individuals from Brazil, Guyana and Suriname, with an intraspecific genetic distance of 1.41%. One individual from Ecuador, whose species was not defined in GenBank, belonged to a distinct lineage (clade E). Two other specimens without species level-definition were grouped with *Oecomys concolor* (branch F), with a genetic distance of 0.79%. One other individual (clade G), also identified as *Oecomys concolor* in GenBank, exhibited a distinct lineage, showing a large genetic distance (12.88%) from branch F. All *Oecomys roberti* specimens were grouped together (clade D), with a genetic distance of 0.39%. *Oecomys bicolor* formed three clades (A, B and C) with large genetic distances: individuals from the Guyanas and Suriname were grouped together with high support, forming a moderately supported clade (C) with an individual from the Central Amazon (INPA 6775). *Oecomys bicolor* and *Oecomys* sp. from Ecuador and the Negro river (INPA 6770) formed a group with moderate-to-high support (clade B). One individual from the Purus River (clade C) showed a highly supported association with clade B, with a genetic distance of 7.12%. The genetic distance between clades A and B was 8.4%, and that between A and C was 9.89%. *Oecomys rex* also formed two clades (K and L), with a large genetic distance between them (11.92%).

## Discussion

The identification of Rodentia species is often difficult using morphological criteria alone (Granjon et al. 2002, Lecompte et al. 2005, Ben Faleh et al. 2010). Such difficulties are evident in this order mainly because of the existence of cryptic species (Granjon et al. 2002, Musser and Carleton 2005, Lecompte et al. 2005) and new species are continually described (Helgen 2005, Musser et al. 2005). Species identification via molecular methods, such as molecular barcoding using a short genetic marker (Hebert et al. 2003), has been proposed to overcome some of the weaknesses of the traditional approach, which will aid non-taxonomists by fulfilling the urgent requirement for rapid and accurate species identification tools (Teletchea 2010). This approach is potentially useful in the study of rodents (Borisenko et al. 2008, Tamrin and Abdullah 2011, Barbosa 2013). In the present work, employing COI sequences as a tool for species identification was shown to be satisfactory, as the obtained distance patterns provided sufficient information for the identification of specimens whose taxonomic identification at the species level is not straightforward. Most of the species were recovered as monophyletic groups.

The available chromosomal data for *Oecomys* species consist mostly of descriptions of diploid and fundamental numbers, which restricts comparisons with the data obtained in the present work (Table 1). However, high karyotypic diversity can be observed, with countless chromosomal rearrangements between *Oecomys* species being responsible for this diversity. Neither of the two *Oecomys auyantepui* karyomorphs reported in this work had been previously described in the literature. Both individuals (INPA 6751, INPA 6754) showing these two karyomorphs were captured on the same bank (right) of the Jatapú river, approximately 1 km from each other. The karyomorphs only exhibited one ITS, located on X chromosome. ITSs have been observed in other rodents as well (Castiglia et al. 2007, Rovatsos et al. 2011, Suárez-Villota et al. 2013). Short telomeric sequences (TTAGGG)_n_ have been primarily classified as components of satellite DNA (Adegoke et al. 1993). These sequences may be located in subtelomeric and interstitial chromosome positions (Garrido-Ramos et al. 1998) and are subjected to amplification (Arnason et al. 1998, Castiglia et al. 2006). They may also appear during the double-stranded DNA nick repair process (Nergadze et al. 2004, 2007). However, the most commonly accepted scenario is that ITSs signal recent chromosomal rearrangements, such as the transposition of functional telomeric sequences to an interstitial position (Dobigny et al. 2003, Zhdanova et al. 2005), or chromosome fusion events, with the latter being the main source of ITSs in many organisms (Lee et al. 1993, Slijepcevic 1998). Nevertheless, it was not possible to determine the occurrence of either an increase in the diploid number from 2n=64 as a result of a fission event or a decrease from 2n=66 due to a fusion event.

Establishing the evolutionary direction of chromosomal rearrangements is not always possible because most of the available painting data for the Sigmodontinae group are incomplete, and it is not possible to draw definitive conclusions regarding the composition of a putative Sigmodontinae ancestral karyotype (Romanenko et al. 2012). The same is true for *Oecomys*, where it cannot be determined whether the diploid number has increased or decreased because the *in situ* hybridization method used in this study likely does not detect very short (< 1 kb) stretches of (TTAGGG)n sequences. Thus, even if chromosome fusions that would result in a decrease in diploid number have occurred, the fused chromosomes will not always possess an ITS, which may have been lost prior to the fusion or been subjected to molecular erosion (Mandrioli et al. 1999).

Both *Oecomys auyantepui* karyomorphs exhibit similar, predominantly centromeric, subtle heterochromatic blocks. Their NORs are also similar, with three different labeled sites being observed on the same chromosome pairs. The largest chromosomes of both karyomorphs are homologous - those carrying 5S rDNA loci in particular - sharing the same chromosomal region (subtelocentric chromosomes), position (long arm, proximal) and number of labeled sites, as inferred based on the increased resolution provided by G-banding. Thus, much like the NOR-carrying pairs, these chromosomes were not involved in chromosomal alteration processes leading to the occurrence of two different diploid numbers in *Oecomys auyantepui*. Mitochondrial DNA analysis grouped all *Oecomys auyantepui* specimens onto a single branch (Fig. 6) with a high support value and low intraspecific genetic distance (1.41%), indicating that the occurrence of these two karyomorphs may be due to chromosomal polymorphism and not to the existence of two differentiated evolutionary units, as the intraspecific genetic distance is consistent with available data for other Sigmodontinae and the family Cricetidae in general (Smith and Patton 1993, Patton 1999,Ventura 2009).

Current phylogenetic and karyotypic data suggest the existence of a complex of *Oecomys bicolor* species (Smith and Patton 1999, Flores 2010, Andrade and Bonvincino 2003). Four different diploid numbers have previously been characterized in the Brazilian Amazon, varying from 54 to 86 chromosomes, with 2n=80 being the most common (Gardner and Patton 1976, Patton et al. 2000, Andrades-Miranda et al. 2000, Andrades-Miranda et al. 2001, Lira 2012). Comparison of the karyotypic patterns of *Oecomys bicolor* captured along the Jatapú and Purus rivers revealed a similar chromosomal organizational pattern for individuals with 2n=80 chromosomes. However, the karyotypic pattern of individuals collected on the banks of the Jari river diverges, with a diploid number 2n=82 and FN=116 (Lira 2012). The NORs described in the present work (7 labeled sites) occurred in larger numbers than what had been previously described for the species (1 to 4 labeled sited) (Andrades-Miranda 2001, Lira 2012). These NORs do not refer to the labeling of acidic heterochromatic regions, as fluorescent *in situ* hybridization using 18S rDNA probes revealed the existence of twelve chromosome pairs carrying these sequences. A larger number of sites compared with the number identified through silver nitrate staining, which is a common occurrence and is observed in other groups (Lira 2012). This disparity stems from the fact that the latter technique labels proteins associated with the nucleolar structure and not ribosomal DNA regions, thus identifying only NORs that had been active in the preceding interphase (Miller et al. 1976). Thus, the difference in silver-stained sites between different populations may stem from the activity of ribosomal RNA genes. Because rDNA sequence hybridization had not been performed in individuals from the analyzed populations in previous studies, this hypothesis cannot be verified. In contrast, the heterochromatin distribution pattern is similar, with centromeric blocks extending to the short arms of the majority of metacentric and submetacentric chromosomes and both sex chromosomes.

The diploid number determined for *Oecomys rutilus* (2n=54, first described in Lira (2012) did not vary, regardless of the collection site, and no variations in karyotypic structure were observed in the present work. However, the three specimens described previously (Lira, 2012) exhibited differences in their autosomal fundamental number (82, 84 and 86). Such variation may be related to karyotype interpretation, given that it depends on the quality of chromosome preparations, DNA compaction patterns, size and number of chromosomes and errors in the measurement of chromosomal arms. The C-banding pattern observed in *Oecomys rutilus* consisted of very subtle labeling on the majority of chromosomes but was consistent with the expected locations previously described for other *Oecomys* species and the tribe Oryzomyini (Yonenaga-Yassuda et al. 1987, Svartman and Almeida 1992, Silva and Yonenaga-Yassuda 1998, Aniskin and Volobouev 1999, Volobouev and Aniskin 2000, Andrades-Miranda et al. 2002, Bonvicino et al. 2005, Lira 2012).

Based on the amplitude of the genus distribution, Langguth et al. (2005) suggested *Oecomys catherinae* (2n=60) as the ancestral taxon; the same finding was reported by Weksler (2006), based on phylogenetic analysis of the IRBP gene and morphological data for *Oecomys bicolor*, *Oecomys catherinae*, *Oecomys concolor*, *Oecomys mamorae* and *Oecomys trinitatis*. Although the current phylogenetic analysis based on COI sequences was limited to a single marker and did not consider several of the taxa analyzed by Flores (2010), it showed similar results with high support values, such as monophyly of the genus *Oecomys*, which was also observed in molecular studies using other markers (Smith and Patton 1999, Weksler 2006). However, considering *Oecomys rex* as a sister group of *Oecomys catherinae*, which would classify both species as ancestral taxa (Flores 2010), the basal diploid number would be approximately 60/62 chromosomes. Therefore, it must be noted that molecular analyses did not detect an increasing or decreasing tendency in the diploid number between the branches, suggesting a complex karyotypic structure, as shown by the different diploid numbers obtained for the same morphological species. Moreover, the phylogenetic analysis placed all individuals in a single group.

In the present work, NORs were found to be preferentially located in the terminal regions of chromosomes, and their number increased with the diploid number; this pattern is also present in other members of the family Cricetidae (Lira 2012, Romanova et al. 2006, Ventura 2009, Fagundes et al. 1997). These data agree with FISH results obtained using the 18S ribosomal DNA probe, confirming the presence of two labeled pairs for *Oecomys auyantepui* and *Oecomys rutilus*. Labeling of four NORs was observed in *Oecomys rutilus*, whereas three were detected in *Oecomys auyantepui*. Lira (2012) described four labeled sites in *Oecomys rex*, again suggesting that it may constitute a basal taxon. In *Oecomys bicolor*, five chromosome pairs exhibited labeling, though not all displayed labeling on both homologous chromosomes. The multiple 18S rDNA sites observed in *Oecomys bicolor* likely derive from duplication and dispersion. Di Meo et al. (1993) reported that the difference in the NOR distribution in correlated species is ascribed to rearrangements that have accumulated since the divergence of the common ancestor, mainly via inversions and Robertsonian translocations. Grozdanov et al. (2003) and Britton-Davidian et al. (2012) stated that NOR diversity among rodents is an indicator of high intrachromosomal transposition rates in the absence of visible rearrangements, suggesting, once again, that this character represents a derived state for this taxon. Despite this fact, the interstitial position of 5S rDNA is related to sequence protection, thereby avoiding possible crossing-over or transposition events, which are more frequent in terminal regions (Martins and Galetti Jr 1999). This scenario is made evident by comparing the degree of conservation in the position and location of this sequence compared with 45S rRNA. Ventura et al. (2012) described a similar situation in Akodontini, which shows conservation of 5S rDNA chromosomal sites, despite large chromosomal variability within the group.

*Oecomys* species have undergone intense chromosomal alteration processes, as confirmed by the observed karyotypic patterns, indicating high local diversity and an ample distribution for the taxa under study. However, the limited taxonomic sample available, in terms of both *Oecomys* individuals and molecular data renders the determination of which evolutionary processes have led to the variability in karyotype morphology more difficult. Furthermore, the current data reinforce the necessity for integrative taxonomy, where genetic tools should be used in conjunction with morphological analysis to delimit *Oecomys* taxa.

## Conclusions

The intra- and interspecific variations observed in the diploid number of *Oecomys* species indicate that chromosomal rearrangements such as fusions/fissions, translocations and duplications have led to the appearance of different diploid numbers and karyotypic formulas. However, telomere sequence hybridization was not found to be a good indicator of autosomal chromosome rearrangements in the *Oecomys* species under study, as no autosomal ITSs could be observed. *Oecomys bicolor*, which is considered to be a derived taxon of the genus (Flores 2010), exhibits the highest diploid number, possibly arising from chromosomal fission events that occurred during its evolutionary history.

## Appendix 1

**Table:** COI sequences of *Oecomys* deposited in GenBank. The voucher, species and collection sites are listed.

Species	Genbank n°	Collection sites
*Oecomys auyantepui*	JQ601277.1	Suriname – Sipaliwini river
	JQ601224.1	Suriname – Sipaliwini river
	JQ601188.1	Suriname – Kutari River
	JQ601163.1	Suriname – Kutari River
	JQ601162.1	Suriname – Kutari River
	JQ601061.1	Suriname: Brownsberg Nature Park
	JQ601060.1	Suriname: Brownsberg Nature Park
	JQ601049.1	Suriname: Brownsberg Nature Park
	HQ545642.1	Suriname: Sipaliwini River
	HQ545608.1	Suriname
	HQ545608.1	Suriname
	JF491641.1	Guiana: Upper Demerara-Berbice, West Pibiri, Mabura
	JF491640.1	Guiana: Upper Takutu-Upper Essequibo
	JF491639.1	Guiana: Upper Takutu-Upper Essequibo
	JF491638.1	Guiana: Potaro-Siparuni
	JF491637.1	Guiana: Potaro-Siparuni
	JF491635.1	Guiana: Cuyuni-Mazaruni
	JF491634.1	Guiana: Cuyuni-Mazaruni
*Oecomys bicolor*	JQ601078.1	Equador: Parque Nacional Yasuni
	JF491655.1	Equador: Napo, Parque Nacional Yasuni
	JF491652.1	Equador: Orellana, Onkone Gare
	JF491649.1	Guiana: Demerara-Mahaic
	JF491648.1	Guiana: Barima-Waini
	JF491647.1	Guiana: Barima-Waini
	JF491644.1	Guiana: Upper Takutu-Upper Essequibo
	JF491643.1	Guiana: Potaro-Siparuni
	JF491642.1	Guiana: Potaro-Siparuni
	JF444363.1	Equador: Orellana
	EU096822.1	Suriname: Sipaliwini
	EU096821.1	Suriname: Sipaliwini
*Oecomys concolor*	JF491662.1	Equador: Napo, Parque Nacional Yasuni
	JF491661.1	Equador: Orellana, Onkone Gare
	JF444368.1	Equador: Orellana
*Oecomys rex*	JF491660.1	Guiana: Potaro-Siparuni
	JF491659.1	Guiana: Potaro-Siparuni
	JQ601068.1	Guiana: 40 Km NE of Surama
	HQ919650.1	Suriname
*Oecomys roberti*	JF491663.1	Guiana: Potaro-Siparuni
*Oecomys rutilus*	JQ601181.1	Suriname: Kutari River Camp
	JF491682.1	Guiana: Potaro-Siparuni
	JF491681.1	Guiana: Potaro-Siparuni
	JF491673.1	Guiana: Potaro-Siparuni Kabukalli Landing, Iwokrama Forest
	JF491671.1	Guiana: Siparuni river
	JF491670.1	Guiana: Barima-Waini, Baramita, Old World
	JF491667.1	Guiana: Barima-Waini, Baramita, Old World
	JF491665.1	Guiana: Upper Takutu-Upper Essequibo
	EU095454.1	Guiana: Upper Demerara-Berbice
	EU095453.1	Equador: Napo
*Oecomys* sp.	JF491619.1	Equador: Napo, Parque Nacional Yasuni

## References

[B1] Adegoke JA Arnason U Widegren B (1993) Sequence organization and evolution, in all extant whalebone whales, of a DNA satellite with terminal chromosome localization. Chromosoma 102: 382–388. doi: 10.1007/BF00360402 8365348 10.1007/BF00360402

[B2] Andrade AFB Bonvincino CR (2003) A new karyological variant of *Oecomys* (Rodentia: Sigmodontinae) and its phylogenetic relationship based on molecular data. Genome 46: 195–203. doi: 10.1139/g02-123 12723035 10.1139/g02-123

[B3] Andrades-Miranda J Zanchin NIT Oliveira LFB Langguth A Mattevi MS (2000) Cytogenetic studies in nine taxa of the genus *Oryzomys* (Rodentia, Sigmodontinae) from Brazil. Mammalia 65: 461–472. doi: 10.1515/mamm.2001.65.4.461

[B4] Andrades-Miranda J Oliveira LFB Zanchin NIT Mattevi MS (2001) Chromosome studies of seven species of *Oligoryzomys* (Rodentia: Sigmodontinae) from Brazil. Journal of Mammalogy 82(4): 1080–1091. doi: 10.1644/1545-1542(2001)082<1080:CSOSSO>2.0.CO;2

[B5] Andrades-Miranda J Zanchin NIT Oliveira AR Langguth A Mattevi MS (2002) (TTAGGG)n telomeric sequence hybridization indicating centric fusion rearrangements in the karyotype of the rodent *Oryzomys subflavus*. Genetica 114: 11–16. doi: 10.1023/A:1014645731798 11990754 10.1023/a:1014645731798

[B6] Aniskin VM Volobouev VT (1999) Comparative chromosome banding of two South-American species of rice rats of the genus *Oligoryzomys* (Rodentia: Sigmodontinae). Chromosome Research 7: 557–562. doi: 10.1023/A:1009245729902 10598571 10.1023/a:1009245729902

[B7] Arnason U Alderdice PW Lien J Widegren B (1998) Highly repetitive DNA in the baleen whale genera *Balaenoptera* and *Megaptera*. Journal of Molecular Evolution 27: 217–221. doi: 10.1007/BF02100077

[B8] Asfora PH Palma ART Astúa D Geise L (2011) Distribution of *Oecomys catherinae* Thomas, 1909 (Rodentia: Cricetidae) in northeastern Brazil with karyotypical and morphometrical notes. Biota Neotropica 11(2): 415–424. doi: 10.1590/S1676-06032011000200039

[B9] Baker RJ Koop BF Haiduk MW (1983) Resolving systematic relationship with G-bands: a study of five genera of South American Cricetide Rodents. Systematic Zoology 32(4): 403–416. doi: 10.2307/2413167

[B10] Barbosa S Pauperio J Searle JB Alves PC (2013) Genetic identification of Iberian rodent species using both mitochondrial and nuclear loci: application to noninvasive sampling. Molecular Ecology Resources 13: 43–56. doi: 10.1111/1755-0998.12024 23095787 10.1111/1755-0998.12024

[B11] Ben Faleh AR Cosson JF Tatard C Ben Othmen Said AK Granjon L (2010) Are there two cryptic species of the lesser Jerboa *Jaculus jaculus* (Rodentia, Dipodidae) in Tunisia? Evidence from molecular, morphometric, and cytogenetic data. Biological Journal of Linnaean Society 99: 673–686. doi: 10.1111/j.1095-8312.2010.01374.x

[B12] Bonvicino CR Otazú IB Vilela JF (2005) Karyologic and molecular analysis of *Proechimys* Allen, 1899 (Rodentia, Echimyidae) from the Amazonian region. Arquivos do Museu Nacional do Rio de Janeiro 63(1): 191–200.

[B13] Bonvicino CR Oliveira JÁ D’Andrea OS (2008) Gênero *Oecomys*. In: Bonvicino CR de Oliveira JA D'Andrea PS (Eds) Guia dos Roedores do Brasil: Com chaves para gêneros baseadas em caracteres externos. Centro Pan-Americano de Febre Aftosa – OPAS/OMS, Rio de Janeiro, 46–47.

[B14] Borisenko AV Lim BK Ivanova NV Hanner RH Hebert PDN (2008) DNA barcoding in surveys of small mammal communities: a field study in Suriname. Molecular Ecology Resources 8: 471–479. doi: 10.1111/j.1471-8286.2007.01998.x 21585824 10.1111/j.1471-8286.2007.01998.x

[B15] Britton-Davidian J Robinson TJ Veyrunes F (2012) Systematics and evolution of the African pygmy mice, subgenus Nannomys: a review. Acta Oecologica 42: 41–49. doi: 10.1016/j.actao.2012.01.001

[B16] Carleton MD Musser GG (1984) Muroid rodents. In: Anderson S Jones Jr JK (Eds) Orders and Families of Recent Mammals of the World. John Wiley and Sons, New York, 289–379.

[B17] Carleton MD Emmons LH Musser GG (2009) A new species of the rodent genus *Oecomys* (Cricetidae: Sigmodontinae: Oryzomyini) from Eastern Bolivia, with emended definitions of *O. concolor* (Wagner) and *O. mamorae* (Thomes). American Museum of Natural History 3661: 1–32. doi: 10.1206/612.1

[B18] Castiglia R Garagna S Merico V Oguge N Corti M (2006) Cytogenetics of a new cytotype of african Mus (subgenus Nannomys) minutoides (Rodentia, Muridae) from Kenya: C- and G- banding and distribution of (TTAGGG)n telomeric sequences. Chromosome Research 14: 587–594. doi: 10.1007/s10577-006-1054-5 16823620 10.1007/s10577-006-1054-5

[B19] Castiglia R Makundi R Corti M (2007) The origin of an unusual sex chromosome constitution in *Acomys* sp. (Rodentia, Muridae) from Tanzania. Genetica 131: 201–207. doi: 10.1007/s10709-006-9127-0 17180438 10.1007/s10709-006-9127-0

[B20] Di Meo GP Ianuzzi L Perucatti A Ferrara L (1993) Identification of nucleolus organizer chromosomes in sheep (*Ovis aries* L.) by sequential GBG/Ag-NOR and RBG/Ag-NOR techniques. Cytobios 75: 183–190. 7694824

[B21] Dobigny G Ozouf-Costaz C Bonillo C Volobouev V (2003) Evolution of rRNA gene clusters and telomeric repeats during explosive genome repatterning in *Taterillus* X (Rodentia, Gerbillinae). Cytogenetic Genome Research 103: 94–103. doi: 10.1159/000076296 15004471 10.1159/000076296

[B22] Emmons LH Feer F (1997) Neotropical rainforest mammals: a field guide. University of Chicago Press, Chicago, 307 pp.

[B23] Fagundes V Vianna-Morgante AM Yonenaga-Yassuda Y (1997) Telomeric sequences localization and G-banding patterns in the identification of a polymorphic chromosomal rearrangement in the rodent *Akodon cursor* (2n= 14, 15 and 16). Chromosome Research 5: 228–232. doi: 10.1023/A:1018463401887 9244449 10.1023/a:1018463401887

[B24] Flores TA (2010) Diversidade morfológica e molecular do gnênero Oecomys Thomas, 1906 (Rodentia: Cricetidae) na Amazônia oriental brasileira. PhD Thesis, Universidade de Federal do Pará, Museu paraense Emílio Goeldi, 102 pp.

[B25] Ford CE Hamerton JL (1956) The chromosomes of man. Nature 178: 1020–1023. doi: 10.1038/1781020a0 13378517 10.1038/1781020a0

[B26] Gardner AL Patton JL (1976) Karyotypic variation in oryzomyinae rodents (Cricetinae) with comments on chromosomal evolution in the Neotropical cricetinae complex. Occasional Papers of the Museum of Zoology, Louisiana State University 49: 1–48.

[B27] Garrido-Ramos MA de la Herran R Rejon CR Rejon MR (1998) A satellite DNA of the Sparidae Family (Pisces, Perciformes) associated with telomeric sequences. Cytogenetics Cell Genetics 83: 3–9. doi: 10.1159/000015151 9925909 10.1159/000015151

[B28] Granjon L Aniskin VM Volobouev V Sicard B (2002) Sand-dwellers in rocky habitats: a new species of *Gerbillus* (Mammalia: Rodentia) from Mali. Journal of Zoology 256: 181–190. doi: 10.1017/S0952836902000213

[B29] Gross MC Schneider CH Valente GT Martins C Feldberg E (2010) Variability of 18S rDNA locus among Symphysodon fishes: chromosomal rearrangements. Journal of Fish Biology 76: 1117–1127. doi: 10.1111/j.1095-8649.2010.02550.x 20409165 10.1111/j.1095-8649.2010.02550.x

[B30] Grozdanov P Geogiev O Karagyozov L (2003) Complete sequence of the 45-kb mouse ribosomal DNA repeat: analysis of the intergenic spacer. Genomics 82: 637–643. doi: 10.1016/S0888-7543(03)00199-X 14611805 10.1016/s0888-7543(03)00199-x

[B31] Hall T (2001) Bioedit version 5.0.6. Department of Microbiology, North Carolina State University Raleigh.

[B32] Hebert PDN Cywinska A Ball SL de Waard JR (2003) Biological identifications through DNA barcodes. Proceedings of the Royal Society 270: 313–321. doi: 10.1098/rspb.2002.2218 10.1098/rspb.2002.2218 PMC1691236 12614582

[B33] Howell WM Black DA (1980) Controlled silver staining of nucleolus organizer regions with a protective colloidal developer: a 1-step method. Experientia 3: 1014–1015. doi: 10.1007/BF01953855 10.1007/BF01953855 6160049

[B34] Helgen KM (2005) A new species of murid rodent (genus *Mayermys*) from south-eastern New Guinea. Mammalian Biology 70: 61–67. doi: 10.1078/1616-5047-00176

[B35] Ivanova NV Zemlak TS Hanner RH Hebert PDN (2007) Universal primer cocktails for fish DNA barcoding. Molecular Ecology Notes 4: 544–548. doi: 10.1111/j.1471-8286.2007.01748.x

[B36] Ijdo JW Wells RA Baldini A Reeders ST (1991) Improved telomere detection using a telomere repeat probe (TTAGGG)n generated by PCR. Nucleic Acids Research 19(17): . doi: 10.1093/nar/19.17.4780 10.1093/nar/19.17.4780 PMC328734 1891373

[B37] Langguth A Maia V Mattevi MS (2005) Karyology of large size brazilian species of the genus *Oecomys* Thomas, 1906 (Rodentia, Muridae, Sigmodontinae). Arquivos do Museu Nacional 63(1): 183–190.

[B38] Lecompte E Brouat C Duplantier JM Galan M Granjon L Loiseau A Mouline K Cosson JF (2005) Molecular identification of four cryptic species of *Mastomys* (Rodentia, Murinae). Biochemical Systematics and Ecology 33: 681–689. doi: 10.1016/j.bse.2004.12.015

[B39] Lee C Sasi R Lin CC (1993) Interstitial localization of telomeric DNA sequences in the Indian muntjac chromosomes: further evidence for tandem chromosome fusions in the karyotypic evolution of the Asian muntjacs. Cytogenetics and Cell Genetics 63: 156–159. doi: 10.1159/000133525 8485991 10.1159/000133525

[B40] Levan A Fredga K Sandber AA (1964) Nomenclature for centromeric position on chromosomes. Hereditas 52: 201–220. doi: 10.1111/j.1601-5223.1964.tb01953.x

[B41] Lira T (2012) Citogenética clássica e molecular de alguns representantes da tribo Oryzomyini (Rodentia, Cricetidae) da Amazônia Central. Dissertation, Instituto Nacional de Pesquisas da Amazônia, 84 pp.

[B42] Mandrioli M Cuoghi B Marini M Manicardi GC (1999) Localization of the (TTAGGG)n telomeric repeat in the chromosome of the pufferfish *Tetraodon fluviatilis* (Hamilton Buchanan) (Osteichthyes). Caryologia 52: 155–157. doi: 10.1080/00087114.1998.10589167

[B43] Martins C Galetti PM (1999) Chromosomal localization of 5s rDNA genes in *Leporinus* fish (Anostomidae, Characiformes). Chromosome Research 7: 363–367. doi: 10.1023/A:1009216030316 10515211 10.1023/a:1009216030316

[B44] Miller DA Dev VG Tantravahi R Miller OJ (1976) Suppression of human nucleolus organizer activity in mouse–human somatic hybrid cells. Experimental Cell Research 101: 235–243. doi: 10.1016/0014-4827(76)90373-6 61125 10.1016/0014-4827(76)90373-6

[B45] Musser GG Carleton MD (2005) Superfamily Muroidea. In: Wilson DE Reeder DA (Eds) Mammal species of the world: a taxonomic and geographic reference. Johns Hopkins University Press, Baltimore, Maryland, 43–79.

[B46] Nergadze SG Rocchi M Azzalin CM Mondello C Giulotto E (2004) Insertion of telomeric repeats at intrachromosomal break sites during primate evolution. Genome Research 14: 1704–1710. doi: 10.1101/gr.2778904 15310657 10.1101/gr.2778904 PMC515315

[B47] Nergadze SG Santagostino MA Salzano A Mondello C Giulotto E (2007) Contribution of telomerase RNA retrotranscription to DNA double-strand break repair during mammalian genome evolution. Genome Biology 8: . doi: 10.1186/gb-2007-8-12-r260 10.1186/gb-2007-8-12-r260 PMC2246262 18067655

[B48] Patton JL (1999) Family Geomyidae. In: Alvarez STC Patton JL (Eds) Los Mamíferos del Noroeste. Centro de Investigaciones Biologicas del Noroeste, La Paz, 321–350.

[B49] Patton JL da Silva MNF Malcolm JR (2000) Mammals of the rio Juruá and the evolutionary and ecological diversification of Amazonia. American Museum of Natural History 244: 1–306. doi: 10.1206/0003-0090(2000)244<0001:MOTRJA>2.0.CO;2

[B50] Patton JL Sherwood SW (1983) Chromosome evolution and speciation in rodents. Annual Review of Ecology and Systematics 14: 139–159. doi: 10.1146/annurev.es.14.110183.001035

[B51] Pinheiro PS Geise L (2008) Non-volant mammals of Picinguaba, Ubatuba, state of São Paulo, southeastern Brazil. Boletim do Museu de Biologia Mello Leitão 23: 51–59.

[B52] Pinkel D Straume T Gray JW (1986) Cytogenetic analysis using quantitative, high sensitivity, fluorescence hybridization. Proceeding of the National Academy of Sciences 83: 2934–2938. doi: 10.1073/pnas.83.9.2934 10.1073/pnas.83.9.2934 PMC323421 3458254

[B53] Prado JR Percequillo AR (2013) Geographic distribution of the genera of the Tribe Oryzomyini (Rodentia: Cricetidae: Sigmodontinae) in South America: patterns of distribution and diversity. Arquivos de Zoologia 44: 1–120.

[B54] Romanova LG Anger M Zatsepina OV Schultz RM (2006) Implication of nucleolar protein SURF6 in ribosome biogenesis and preimplantation mouse development. Biology of Reproduction 75(5): 690–696. doi: 10.1095/biolreprod.106.054072 16855206 10.1095/biolreprod.106.054072

[B55] Romanenko SA Perelman PL Trifonov VA Graphodatsky AS (2012) Chromosomal evolution in Rodentia. Heredity 108: 4–16. doi: 10.1038/hdy.2011.110 22086076 10.1038/hdy.2011.110 PMC3238120

[B56] Ronquist F Huelsenbeck JP (2003) MrBayes 3: Bayesian phylogenetic inference under mixed models. Bioinformatics 19: 1572–1574. doi: 10.1093/bioinformatics/btg180 12912839 10.1093/bioinformatics/btg180

[B57] Rosa CC Flores T Pieczarka JC Rossi RV Sampaio MIC Rissino JD Amaral PJS Nagamachi CY (2012) Genetic and morphological variability in South American rodent *Oecomys* (Sigmodontinae, Rodentia): evidence for a complex of species. Journal of Genetics 91(3): 1–13. doi: 10.1007/s12041-012-0182-2 23271012 10.1007/s12041-012-0182-2

[B58] Rovatsos MT Marchal JA Romero-Fernández I Fernández FJ Giagia-Athanosopoulou EB Sánchez A (2011) Rapid, independente, and extensive amplification of repeats in pericentromeric regions in karyotypes of arvicoline rodents. Chromosome Research 19: 869–882. doi: 10.1007/s10577-011-9242-3 21979796 10.1007/s10577-011-9242-3

[B59] Sambrook J Russell DW (2001) Molecular Cloning: A Laboratory Manual (4th ed). Cold Spring Harbor Press, Cold Spring Harbor, New York, 31 pp.

[B60] Sanger F Nicklen S Coulson AR (1977) DNA sequencing with chain-terminating inhibitors. Proceedings of the National Acadademy of Sciences 74: 5463–5467. doi: 10.1073/pnas.74.12.5463 10.1073/pnas.74.12.5463 PMC431765 271968

[B61] Santos MS (2010) Mapeamento gênico de sítios de DNAr 5S e 18S em Astyanax scabripinnis (Characiformes, Characidae). Dissertação de mestrado, USP, São Paulo, 136 pp.

[B62] Seabrigth M (1971) A rapid banding technique for human chromosomes. Lancet 2: 971–972. doi: 10.1016/S0140-6736(71)90287-X 10.1016/s0140-6736(71)90287-x 4107917

[B63] Silva MJJ Yonenaga-Yassuda Y (1998) Karyotype and chromosomal polymorphism of an undescribed *Akodon* from Central Brazil, a species with the lowest known diploid chromosome number in rodents. Cytogenetics and Cell Genetics 81: 46–50. doi: 10.1159/000015006 9691174 10.1159/000015006

[B64] Slijepcevic P (1998) Telomeres and mechanisms of Robertsonian fusions. Chromosoma 107: 136–140. doi: 10.1007/s004120050289 9601982 10.1007/s004120050289

[B65] Smith MNF Patton JL (1999) Phylogenetic relationship and the radiation of sigmodontine rodents in South America: Evidence from cytochrome *b*. Journal of Mammalian Evolution 6(2): 89–128. doi: 10.1023/A:1020668004578

[B66] Smith MNF Patton JL (1993) The diversification of south American murid rodents: Evidence from mitochondrial DNA sequence data for the akodontine tribe. Biological Journal of the Linnean Society 50: 149–177. doi: 10.1111/j.1095-8312.1993.tb00924.x

[B67] Suárez-Villota E Di-Nizo CB Neves CL Silva MJJ (2013) First cytogenetic information for *Drymoreomys albimaculatus* (Rodentia, Cricetidae), a recently described genus from Brazilian Atlantic Forest. ZooKeys 303: 65–76. doi: 10.3897/zookeys.303.4873 10.3897/zookeys.303.4873 PMC3689069 23794904

[B68] Sumner AT (1972) A simple technique for demonstrating centromeric heterochromatin. Experimental Cell Research 75: 304–306. doi: 10.1016/0014-4827(72)90558-7 4117921 10.1016/0014-4827(72)90558-7

[B69] Svartman M (1989) Levantamento cariotípica de roedores da região do Distrito Federal. Dissertação de Mestrado, Universidade de São Paulo, 160 pp.

[B70] Svartman M Almeida EJC (1992) Comparative karyotypic analysis of two Calomys species (Rodentia, Cricetidae) from Central Brazil. Caryologia 45(1): 35–42. doi: 10.1080/00087114.1992.10797208

[B71] Tamrin NAM Abdullah MT (2011) Molecular phylogenetics and systematics of five genera of Malaysian murine rodents (*Maxomys*, *Sundamys*, *Leopoldamys*, *Niviventer* and *Rattus*) inferred from partial mitochondrial cytochrome *c* oxidse subunit I (COI) gene. Journal of Science and Technology in the Tropics 7: 75–86.

[B72] Tamura K Stecher G Peterson D Filipski A Kumar S (2013) MEGA6: Molecular Evolutionary Genetics Analysis Version 6.0. Molecular Biology and Evolution 30: 2725–2729. doi: 10.1093/molbev/mst197 24132122 10.1093/molbev/mst197 PMC3840312

[B73] Teletchea F (2010) After 7 years and 1000 citations: Comparative assessment of the DNA barcoding and the DNA taxonomy proposals for taxonomists and non- taxonomists. Mitochondrial DNA 21: 206–226. doi: 10.3109/19401736.2010.532212 21171865 10.3109/19401736.2010.532212

[B74] Ventura K (2009) Estudos de citogenética e de filogenia molecular em roedores da tribo akodontini. Tese de doutorado, USP, São Paulo, 164 pp.

[B75] Ventura K Sato-Kuwabara Y Fagundes V Geise L Leite YLR Costa LP Silva MJJ Yonenaga-Yassuda Y Rodrigues MT (2012) Phylogeographic structure and karyotypic diversity of the Brasilian shrew mouse (*Blarinomys breviceps*, Sigmodontinae) in the Atlantic Forest. Cytogenetic and Genome Research 138(1): 19–30. doi: 10.1159/000341887 22907314 10.1159/000341887

[B76] Volobouev VT Aniskin VM (2000) Comparative chromosome banding analysis of three South-American species of rice rats of the genus *Oryzomys* (Rodentia: Sigmodontinae). Chromosome Research 8: 295–304. doi: 10.1023/A:1009223210737 10919720 10.1023/a:1009223210737

[B77] Zhdanova NS Karamisheva TV Minina J Astakhova NM Lansdorp P Kammori M Rubtsov NB Searle JB (2005) Unusual distribution pattern of telomeric repeats in the shrews *Sorex araneus* and *Sorex granarie*s. Chromosome Research 13: 617–625. doi: 10.1007/s10577-005-0988-3 16170626 10.1007/s10577-005-0988-3

[B78] Weksler M (2006) Phylogenetic relationships of the oryzomine rodents (Muroidea: Sigmodontinae): separate and combined analyses of morphological and molecular data. Bulletin of the American Museum of Natural History (New York) 296: 1–149. doi: 10.1206/0003-0090(2006)296[0001:PROORM]2.0.CO;2

[B79] Yonenaga-Yassuda Y Pereira LA L’Abbate M (1887) Chromosomal polymorphism in *Akodon reinhardti* Langguth, 1975 (Rodentia Cricetidae). Revista Brasileira de Genética 10: 199–208.

